# Pharmacokinetics, safety, and tolerability of ceftibuten-ledaborbactam etzadroxil in participants with renal impairment

**DOI:** 10.1128/aac.00860-26

**Published:** 2026-09-28

**Authors:** Carlos Fernando de Oliveira, Kathryn Lowe, Penny Fatato, Thomas Marbury, Karine Litherland, Marc Engelhardt, Kamal A. Hamed, Paul C. McGovern

**Affiliations:** 1 De Oliveira Consulting LLC, New Hope, Pennsylvania, USA; 2 Venatorx Pharmaceuticals, Inc. 540451 , Malvern, Pennsylvania, USA; 3 Orlando Clinical Research Center, Orlando, Florida, USA; 4 Basilea Pharmaceutica International Ltd 449688 , Allschwil, Switzerland; 5 McGovern ID Consulting LLC, Berwyn, Pennsylvania, USA; University of Houston, Houston, Texas, USA

**Keywords:** ceftibuten-ledaborbactam etzadroxil, pharmacokinetics, renal impairment, safety, tolerability

## Abstract

Ledaborbactam etzadroxil is an orally bioavailable prodrug of ledaborbactam, a broad-spectrum β-lactamase inhibitor being developed in combination with ceftibuten for the treatment of complicated urinary tract infections caused by antibiotic-resistant pathogens. As both agents are primarily eliminated via renal excretion, dose adjustment may be required in patients with impaired renal function. This phase 1 study (NCT05488678) evaluated the pharmacokinetics and safety of ceftibuten and ledaborbactam etzadroxil in participants with normal renal function; mild, moderate, or severe renal impairment; or end-stage renal disease (ESRD) undergoing hemodialysis. Participants received a single dose of ceftibuten-ledaborbactam etzadroxil (600 mg/600 mg); those with ESRD received doses both before and after hemodialysis. Among 32 enrolled participants, the mean age was 62.2 years, and 81.3% were male. For both ceftibuten and ledaborbactam, decreasing renal function was associated with increased plasma exposure and reduced renal clearance (CLr). In participants with mild, moderate, or severe renal impairment, plasma exposure of ceftibuten increased 1.3-, 2.3-, and 3.7-fold, respectively, and ledaborbactam increased 1.5-, 1.9-, and 4.4-fold, respectively. Ceftibuten CLr decreased from 2.2 L/h in participants with normal function to 0.3 L/h in those with severe impairment, while ledaborbactam CLr decreased from 3.0 to 0.5 L/h. In participants with ESRD, both drugs were moderately removed (23% and 38% for ceftibuten and ledaborbactam, respectively) by hemodialysis. No deaths, serious adverse events, or treatment-related adverse events leading to study drug discontinuation were reported. These results indicate that dose adjustment of ceftibuten-ledaborbactam etzadroxil will be required for patients with reduced renal function.

This study is registered with ClinicalTrials.gov as NCT05488678.

## INTRODUCTION

Urinary tract infections (UTIs) are among the most common infectious diseases worldwide, affecting an estimated 400 million individuals annually, with the global burden continuing to rise (1–3). Within the United States, the incidence of extended-spectrum β-lactamase (ESBL)-producing bacterial infections, predominantly caused by *Escherichia coli*, increased from 30 to 51 individuals per 10,000 hospital discharges between 2012 and 2020 (4). Resistance among urinary Enterobacterales isolates has similarly increased in the outpatient setting, with ESBL-producing phenotypes rising approximately 30% between 2011 and 2020 (5). Current guidelines recommend fluoroquinolones or trimethoprim-sulfamethoxazole as preferred oral agents for complicated UTIs, including pyelonephritis, caused by ESBL-producing Enterobacterales when susceptibility is confirmed (6). However, resistance rates among ESBL-producing *E. coli* urinary isolates in the United States are approximately 68%–72% to fluoroquinolones and 56% to trimethoprim-sulfamethoxazole. These high resistance rates substantially limit the utility of these drugs and often leave intravenous carbapenems as the standard of care (7). This underscores a critical unmet need for effective oral treatment options that can serve as primary or step-down therapy, thereby reducing the burden of hospitalization and intravenous therapy for patients with UTIs caused by ESBL-producing or multidrug-resistant Enterobacterales.

Ceftibuten is an oral, broad-spectrum, third-generation cephalosporin antibiotic. It exhibits *in vitro* activity against Enterobacterales but is hydrolyzed by ESBLs, limiting its activity against ESBL-producing Enterobacterales (8–10). Ledaborbactam etzadroxil (formerly VNRX-7145) is an inactive, orally bioavailable prodrug of the active moiety ledaborbactam (formerly VNRX-5236), a broad-spectrum β-lactamase inhibitor, that, when combined with ceftibuten, inhibits Ambler class A, C, and D serine β-lactamases, including ESBLs and carbapenemases such as KPC and OXA-48 (11). Urinary excretion is the primary route of elimination for ceftibuten and ledaborbactam, supporting their potential use in the treatment of UTIs (12).

Therefore, a phase 1 study was conducted to evaluate the pharmacokinetics (PK), safety, and tolerability of a single oral dose of ceftibuten-ledaborbactam etzadroxil in participants with renal impairment.

## RESULTS

### Participant demographics and disposition

A total of 32 participants were enrolled and received ceftibuten-ledaborbactam etzadroxil. Thirty-one (96.9%) participants completed the study; one participant in group 5 discontinued because of an unstable hemodialysis port.

Participant demographic and baseline characteristics are summarized in Table 1. The mean age was 62.2 years, ranging from a mean of 50.4 years in group 5 (end-stage renal disease [ESRD]) to 71.2 years in group 4 (severe renal impairment). The majority of participants were male (81.3%), with groups 4 and 5 consisting entirely of male participants. Most participants were White (75.0%) and not Hispanic or Latino (75.0%), and 18.8% were Black/African American; five of the six (83.3%) Black/African American participants enrolled in group 5, constituting 71.4% of group 5. The mean (standard deviation [SD]) body mass index (BMI) of the participants was 29.8 (3.9) kg/m^2^.

### Pharmacokinetics

**TABLE 1:** Participant demographic and baseline characteristics^*a*^^,*b*,*c*^

Parameter	Renal function group					Overall (*N* = 32)
	Group 1: normal function (*n* = 9)	Group 2: mild impairment (*n* = 5)	Group 3: moderate impairment (*n* = 6)	Group 4: severe impairment (*n* = 5)	Group 5: ESRD (*n* = 7)	
Age (years), mean (SD)	60.3 (5.4)	68.4 (4.1)	65.8 (10.1)	71.2 (8.2)	50.4 (7.7)	62.2 (10.0)
Sex at birth, *n* (%)						
Male	8 (88.9)	2 (40.0)	4 (66.7)	5 (100.0)	7 (100.0)	26 (81.3)
Ethnicity, *n* (%)						
Not Hispanic or Latino	7 (77.8)	4 (80.0)	3 (50.0)	4 (80.0)	6 (85.7)	24 (75.0)
Race, *n* (%)						
White	7 (77.8)	5 (100.0)	5 (83.3)	5 (100.0)	2 (28.6)	24 (75.0)
Black or African American	0	0	1 (16.7)	0	5 (71.4)	6 (18.8)
Other	2 (22.2)	0	0	0	0	2 (6.3)
Body weight (kg), mean (SD)	93.2 (7.7)	77.8 (10.1)	86.7 (11.3)	95.2 (9.7)	93.1 (19.8)	89.9 (13.1)
BMI (kg/m^2^), mean (SD)	30.1 (2.8)	28.4 (3.6)	31.1 (3.5)	30.8 (2.8)	28.7 (6.0)	29.8 (3.9)
eGFR (mL/min), mean (SD)	111.6 (14.4)	78.4 (10.6)	43.2 (6.4)	25.4 (2.6)	–^*d*^	–^*d*^
Creatinine clearance (mL/min), mean (SD)	122.8 (28.8)	80.6 (13.6)	55.8 (16.6)	29.4 (6.4)	–^*d*^	–^*d*^

^*a*^ Data include all participants who received at least one dose of ceftibuten-ledaborbactam etzadroxil.

^*b*^ BMI, body mass index; eGFR, estimated glomerular filtration rate; and ESRD, end-stage renal disease.

^*c*^ Normal renal function (eGFR ≥90 mL/min), mild impairment (eGFR 60 to <90 mL/min), moderate impairment (eGFR 30 to <60 mL/min), severe impairment (eGFR <30 mL/min), and ESRD (undergoing standard intermittent dialysis).

^*d*^ Not reported because estimates of renal function were not considered clinically meaningful in participants with ESRD.

#### Participants with normal renal function or mild, moderate, or severe renal impairment

PK parameters were assessed following a single dose of ceftibuten-ledaborbactam etzadroxil. Arithmetic mean (±SD) plasma concentration-time profiles for ceftibuten and ledaborbactam in groups 1–4 are shown in Fig. 1 and 2, respectively. Plasma exposure to both ceftibuten and ledaborbactam was higher in participants with renal impairment than in participants with normal renal function and increased as renal function declined (Fig. 1 and 2).

**Fig 1:**
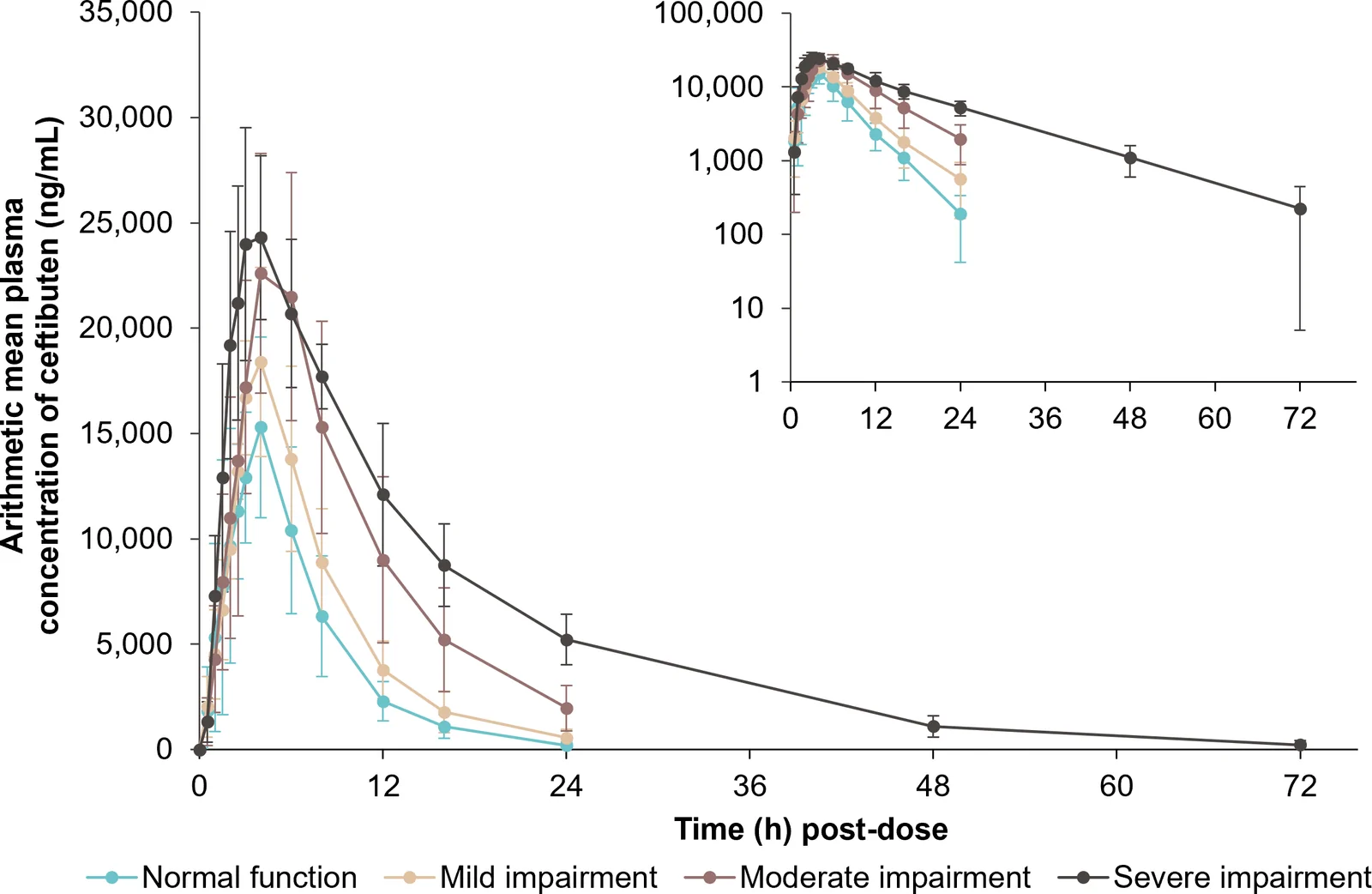
Effects of renal impairment on arithmetic mean (SD) ceftibuten plasma concentrations over time for renal function groups 1–4. Data for the arithmetic mean (±SD) plasma concentrations are shown for all participants who received at least one dose of ceftibuten-ledaborbactam etzadroxil and had at least one evaluable PK sample of any of the assessed analytes. The primary figure shows a linear scale; inset shows a semilogarithmic scale. The lower limit of quantification for ceftibuten is <150 ng/mL. Normal renal function (estimated glomerular filtration rate [eGFR] ≥90 mL/min), mild impairment (eGFR 60 to <90 mL/min), moderate impairment (eGFR 30 to <60 mL/min), and severe impairment (eGFR <30 mL/min). PK, pharmacokinetic.

**Fig 2:**
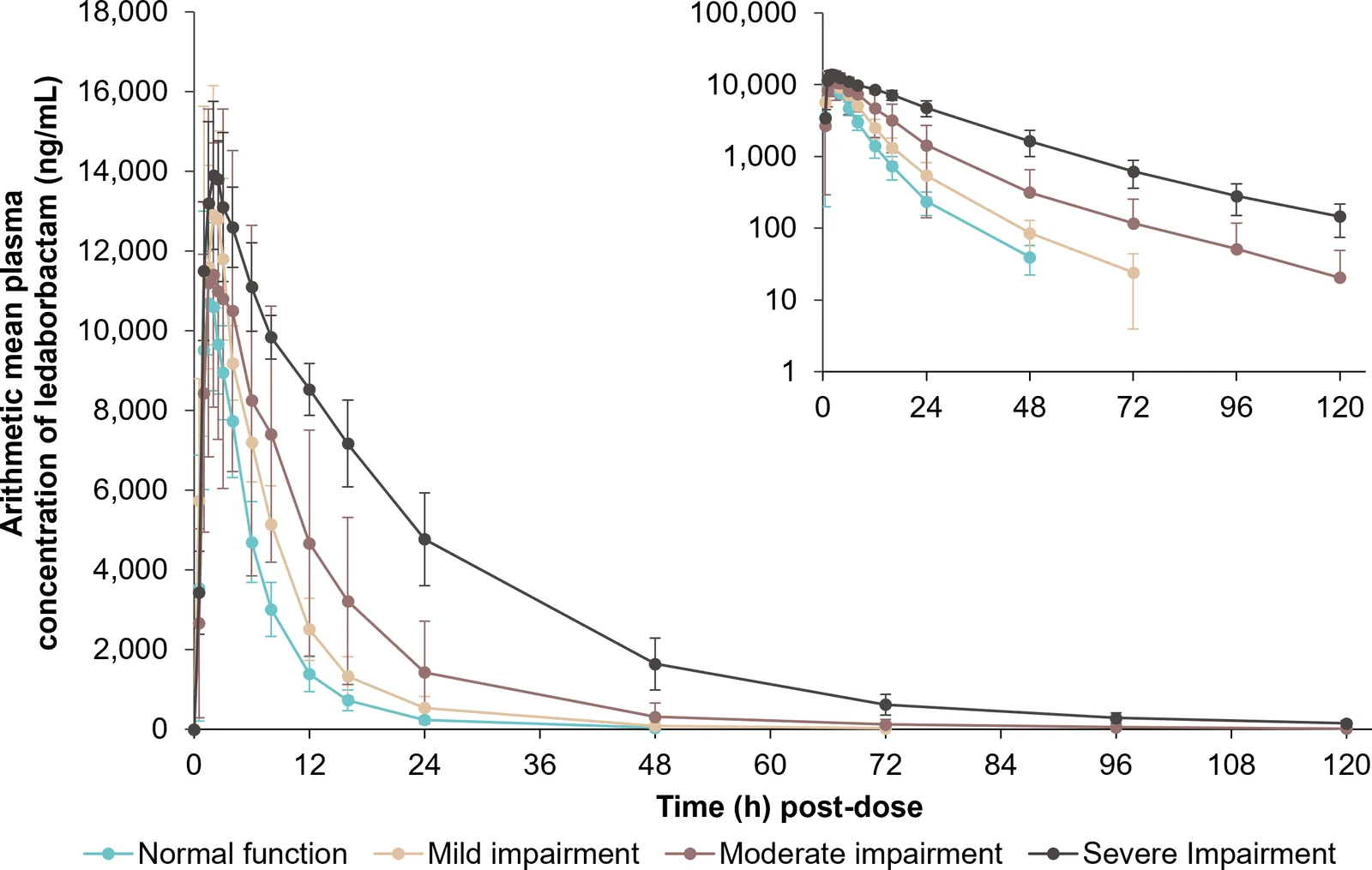
Effects of renal impairment on arithmetic mean (SD) ledaborbactam plasma concentrations over time for renal function groups 1–4. Data for the arithmetic mean (±SD) plasma concentrations are shown for all participants who received at least one dose of ceftibuten-ledaborbactam etzadroxil and had at least one evaluable PK sample of any of the assessed analytes. The primary figure shows a linear scale; inset shows a semilogarithmic scale. The lower limit of quantification of ledaborbactam is <10.0 ng/mL. Normal renal function (estimated glomerular filtration rate [eGFR] ≥90 mL/min), mild impairment (eGFR 60 to <90 mL/min), moderate impairment (eGFR 30 to <60 mL/min), and severe impairment (eGFR <30 mL/min). PK, pharmacokinetic.

The unbound fraction of ledaborbactam in the plasma increased with decreasing renal function across groups 1–4 but remained within the anticipated range of 20%–35% (13). The average of the geometric mean values at 2 and 12 h post-dose was 19.6%, 24.2%, 27.5%, and 27.6% for groups 1–4, respectively.

Linear regression analyses of log-transformed maximum observed plasma concentration (*C*_max_), area under the concentration-time curve from time zero to the last measurable concentration (AUC_0*–t*_), and area under the concentration-time curve from time zero to infinity (AUC_0–∞_) against continuous estimated glomerular filtration rate (eGFR) demonstrated that ceftibuten exposure increased with declining eGFR, consistent with reduced plasma clearance (*P* < 0.0001 for all comparisons; Table 2). Similarly, regression analyses for ledaborbactam showed increasing AUC_0*–t*_ and AUC_0–∞_ with declining eGFR, indicating reduced plasma clearance (*P* < 0.0001 for both comparisons), whereas *C*_max_ was not significantly associated with eGFR. Participant-level log-transformed AUC_0–∞_ values illustrating the inverse relationship between eGFR and systemic exposure for both ceftibuten and ledaborbactam are shown in Fig. 3.

**TABLE 2:** Linear regression analysis of the relationship between plasma PK parameters and eGFR^*a*^^,*b*^

Dependent variable	Regression parameter	Groups 1–4 (*n* = 25)
Ceftibuten		
*C*_max_ (ng/mL)	Intercept	10.3
	Coefficient (90% CI)	−0.0053 (−0.0072,−0.0033)
	*P*-value	<0.0001
	*R* ^2^	0.4902
AUC_0*–t*_ (h∙ng/mL)	Intercept	13.0
	Coefficient (90% CI)	−0.0138 (−0.0163,−0.0112)
	*P*-value	<0.0001
	*R* ^2^	0.7895
AUC_0–∞_ (h∙ng/mL)	Intercept	13.0
	Coefficient (90% CI)	−0.0138 (−0.0163,−0.0113)
	*P*-value	<0.0001
	*R* ^2^	0.7939
Ledaborbactam		
*C*_max_ (ng/mL)	Intercept	9.6
	Coefficient (90% CI)	−0.0019 (−0.0040, 0.0002)
	*P*-value	0.1261
	*R* ^2^	0.0987
AUC_0*–t*_ (h∙ng/mL)	Intercept	12.7
	Coefficient (90% CI)	−0.0142 (−0.0173,−0.0112)
	*P*-value	<0.0001
	*R* ^2^	0.7320
AUC_0–∞_ (h∙ng/mL)	Intercept	12.7
	Coefficient (90% CI)	−0.0143 (−0.0174,−0.0112)
	*P*-value	<0.0001
	*R* ^2^	0.7309

^*a*^ Natural log-transformed PK parameters were modeled as dependent variables, with continuous eGFR as the independent variable, using simple linear regression. Coefficients represent the change in the log-transformed PK parameter per 1 mL/min increase in eGFR. Analyses included all participants who received at least one dose of ceftibuten-ledaborbactam etzadroxil and had at least one evaluable PK sample for the respective analyte.

^*b*^ AUC_0–∞_, area under the concentration-time curve from time zero to infinity; AUC_0*–t*_, area under the plasma concentration-time curve from time zero to the last measurable concentration; CI, confidence interval; *C*_max_, maximum observed plasma concentration; eGFR, estimated glomerular filtration rate; and PK, pharmacokinetic.

**Fig 3:**
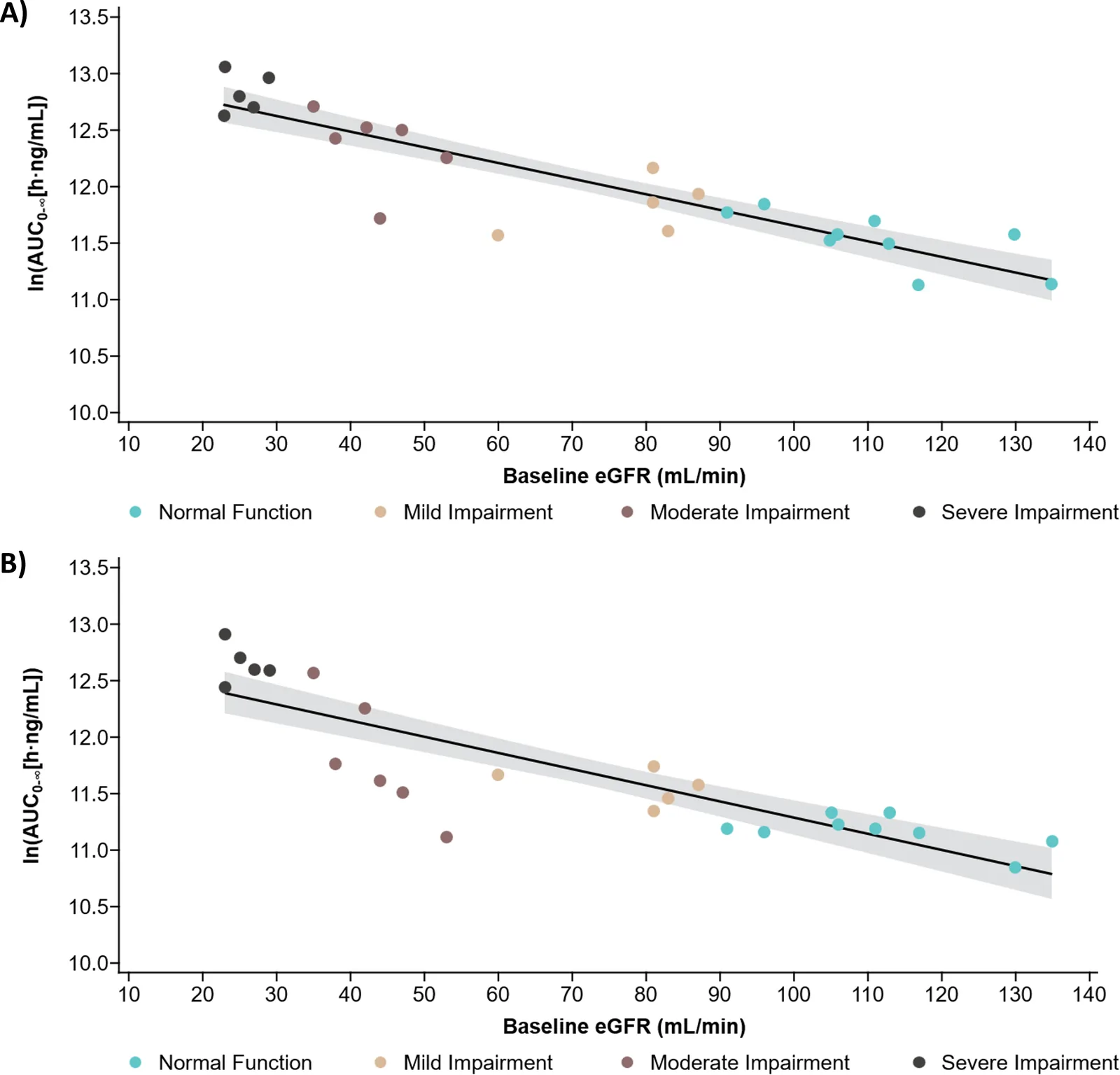
Scatterplots of natural log-transformed AUC_0–∞_ versus baseline eGFR for (**A**) ceftibuten and (**B**) ledaborbactam. Natural log-transformed AUC_0–∞_ values are shown for all participants who received at least one dose of ceftibuten-ledaborbactam etzadroxil and had at least one evaluable PK sample for the respective analyte. Baseline eGFR was calculated on day −2 using the CKD-EPI and converted to mL/min by multiplying the normalized eGFR by the participant’s BSA and dividing by 1.73 m^2^. Linear regression analyses were performed using natural log-transformed AUC_0–∞_ as the dependent variable and continuous baseline eGFR as the independent variable. The fitted regression equations were log(AUC_0–∞_ [h·ng/mL]) = 13.0347 − 0.0138 × eGFR (mL/min) for ceftibuten and log(AUC_0–∞_ [h·ng/mL]) = 12.7202 − 0.0143 × eGFR (mL/min) for ledaborbactam. AUC_0–∞_, area under the concentration-time curve from time zero to infinity; BSA, body surface area; CKD-EPI, Chronic Kidney Diseases Epidemiology Collaboration; eGFR, estimated glomerular filtration rate; and PK, pharmacokinetic.

Plasma PK parameters for ceftibuten and ledaborbactam across renal function groups 1–4 are summarized in Table 3. PK parameters for ledaborbactam etzadroxil across renal function groups 1–4 are summarized in Table S1. Geometric mean AUC_0–∞_ increased approximately 1.3-, 2.3-, and 3.7-fold in participants with mild, moderate, and severe renal impairment (groups 2–4), respectively, compared with participants with normal renal function (group 1). The *C*_max_ and terminal elimination half-life (*t*_1/2_) also increased with decreasing renal function. Plasma clearance of ceftibuten decreased with decreasing renal function; the geometric mean apparent clearance (CL/F) values ranged from 5.90 L/h in group 1 to 1.60 L/h in group 4.

**TABLE 3:** Geometric mean (geometric CV%) and GMR (90% CI) for ceftibuten and ledaborbactam plasma PK parameters across renal function groups 1–4^*a*^^,*b*,*c*^

Renal function group (eGFR range [mL/min])	Ceftibuten			Ledaborbactam		
	*C*_max_ (ng/mL)	*t*_1/2_ (h)	AUC_(0–∞)_ (h∙ng/mL)	*C*_max_ (ng/mL)	*t*_1/2_ (h)	AUC_(0–∞)_ (h∙ng/mL)
Group 1 Normal function (≥90), *n* = 9	16,300 (25.4)	3.3 (14.8)	102,000 (26.2)	11,700 (14.7)	9.0 (27.3)	70,900 (14.7)
Group 2 Mild impairment (60–<90), *n* = 5	19,100 (18.4)	4.0 (17.2)	137,000 (24.6)	13,500 (22.2)	11.5 (32.4)	105,000 (15.8)
GMR (90% CI)	1.17 (0.96, 1.43)	1.21 (1.02, 1.42)	1.35 (1.02, 1.77)	1.16 (0.93, 1.45)	1.28 (0.99, 1.65)	1.48 (1.14, 1.91)
Group 3 Moderate impairment (30–<60), *n* = 6	24,100 (19.8)	5.7 (19.6)	233,000 (35.0)	12,200 (36.1)	16.3 (23.3)	135,000 (56.3)
GMR (90% CI)	1.47 (1.22, 1.78)	1.71 (1.46, 2.00)	2.29 (1.77, 2.97)	1.05 (0.85, 1.29)	1.80 (1.42, 2.30)	1.91 (1.50, 2.44)
Group 4 Severe impairment (<30), *n* = 5	25,200 (20.1)	10.5 (19.0)	375,000 (18.2)	14,700 (9.6)	20.8 (18.6)	315,000 (17.4)
GMR (90% CI)	1.54 (1.26, 1.88)	3.16 (2.69, 3.73)	3.69 (2.81, 4.85)	1.26 (1.01, 1.57)	2.31 (1.79, 2.98)	4.44 (3.43, 5.74)

^*a*^ Data include all participants who received at least one dose of ceftibuten-ledaborbactam etzadroxil and had at least one evaluable PK sample for the respective analyte.

^*b*^ ANOVA, analysis of variance; AUC_0–∞_, area under the concentration-time curve from time zero to infinity; CI, confidence interval; *C*_max_, maximum observed plasma concentration; CV, coefficient of variation; eGFR, estimated glomerular filtration rate; GMR, geometric mean ratio; PK, pharmacokinetic; and *t*_1/2_, terminal elimination half-life.

^*c*^ Data are presented as geometric mean (geometric CV%) unless otherwise specified. GMRs were calculated for groups 2–4 relative to group 1. Statistical analyses were performed using an ANOVA model with log-transformed PK parameters as the response variable and group (groups 1–4, group 5 period 1, and group 5 period 2) as a fixed effect. Estimated mean differences and corresponding 90% CIs were back-transformed to obtain GMRs and their associated 90% CIs.

For ledaborbactam, geometric mean AUC_0–∞_ increased approximately 1.5-, 1.9-, and 4.4-fold in groups 2, 3, and 4, respectively, compared with group 1 (Table 3). The geometric mean *t*_1/2_ also increased with decreasing renal function. CL/F was not calculated for ledaborbactam, as it cannot be derived for a metabolite using noncompartmental analysis.

Urine PK parameters for ceftibuten and ledaborbactam in groups 1–4 are also shown in Table S2. Renal clearance (CLr) decreased with decreasing renal function for both ceftibuten and ledaborbactam. For ceftibuten, geometric mean CLr values ranged from 2.2 L/h in group 1 to 0.3 L/h in group 4, and the geometric mean fraction of dose excreted unchanged into urine (Fe) decreased from 36.5% in group 1 to 19.4% in group 4. For ledaborbactam, geometric mean CLr values ranged from 3.0 L/h in group 1 to 0.5 L/h in group 4, and the geometric mean Fe decreased from 54.6% in group 1 to 36.7% in group 4 (Table S2).

Plasma PK parameters for ledaborbactam etzadroxil in participant groups 1–4 are shown in Table S1. Geometric mean AUC_0–∞_ ranged from 931 to 1,460 h∙ng/mL, and geometric mean CLr ranged from 0.02 L/h for group 1 to 0.006 L/h for group 4.

#### Participants with ESRD on intermittent hemodialysis

Arithmetic mean (±SD) plasma concentration-time profiles for ceftibuten and ledaborbactam, pre- and post-hemodialysis, are shown in Fig. 4. The unbound fraction of ledaborbactam in plasma was highest in group 5 and increased from pre- to post-dialysis. The average geometric mean values at 2 and 12 h post-dose for group 5 pre- and post-dialysis were 30.6% and 31.3%, respectively.

**Fig 4:**
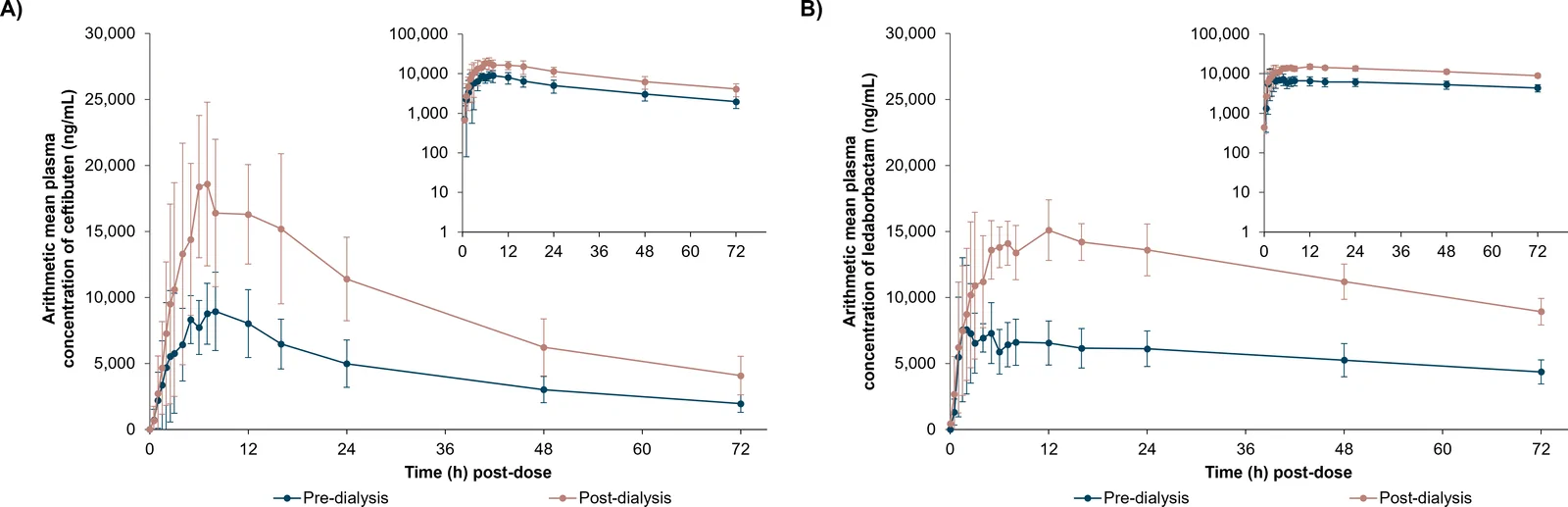
Effects of ESRD on arithmetic mean (±SD) of (**A**) ceftibuten or (**B**) ledaborbactam plasma concentrations over time for renal function group 5. Data for the arithmetic mean (±SD) plasma concentrations are shown for all participants who received at least one dose of ceftibuten-ledaborbactam etzadroxil and had at least one evaluable PK sample of any of the assessed analytes. Primary figure shows linear scale; inset shows semilogarithmic scale. The lower limit of quantification is <150 ng/mL for ceftibuten and <10.0 ng/mL for ledaborbactam. ESRD, end-stage renal disease; PK, pharmacokinetic.

Pre- and post-dialysis plasma PK parameters for ceftibuten and ledaborbactam in participants with ESRD undergoing intermittent hemodialysis (group 5) are shown in Table 4. Ceftibuten geometric mean AUC_0–*t*_ was 52.5% lower when administered prior to hemodialysis compared with administration after completion of hemodialysis. Similarly, ledaborbactam geometric mean AUC_0–*t*_ was 53.8% lower following pre-dialysis administration compared with post-dialysis administration.

**TABLE 4:** Geometric mean (geometric CV%) and GMR (90% CI) for ceftibuten and ledaborbactam plasma PK parameters and HERs in participants with ESRD^*a*^^,*b*,*c*^

PK parameter	Ceftibuten		Ledaborbactam	
	Period 1 pre-dialysis (*n* = 6)	Period 2 post-dialysis (*n* = 6)	Period 1 pre-dialysis (*n* = 6)	Period 2 post-dialysis (*n* = 6)
*C*_max_ (ng/mL)	11,400 (5.3)	21,200 (28.8)	9,980 (37.5)	16,200 (10.2)
GMR (90% CI)	0.70 (0.58, 0.85)	1.30 (1.07, 1.57)	0.85 (0.69, 1.06)	1.39 (1.13, 1.72)
AUC_0–*t*_ (h∙ng/mL)^*d*^	302,000 (29.9)	636,000 (31.2)	391,000 (19.4)	847,000 (8.2)
GMR (90% CI)	3.02 (2.36, 3.87)	6.37 (4.97, 8.16)	5.54 (4.42, 6.94)	11.99 (9.57, 15.01)
AUC_dialysis_ (h∙ng/mL)	25,800 (32.2)	45,400 (58.3)	27,300 (14.9)	45,400 (28.0)
HER1 (*A*_*d*_/dose)	0.23 (41.5)	NA^*e*^	0.38 (13.3)	NA^*e*^
HER2 ([AUC_in_ − AUC_out_]/AUC_in_)	0.43 (18.8)	NA^*e*^	0.33 (13.6)	NA^*e*^

^*a*^ Data include all participants who received at least one dose of ceftibuten-ledaborbactam etzadroxil and had at least one evaluable PK sample for the respective analyte.

^*b*^ *A*_*d*_, amount of drug recovered in dialysate; ANOVA, analysis of variance; AUC_0–∞_, area under the concentration-time curve from time zero to infinity; AUC_0–*t*_, area under the plasma concentration-time curve from time zero to the last measurable concentration; AUC_dialysis_, area under the concentration-time curve during dialysis; AUC_in_, area under the concentration-time curve of arterial concentrations during dialysis; AUC_out_, area under the concentration-time curve of venous concentrations during dialysis; CI, confidence interval; *C*_max_, maximum observed plasma concentration; CV, coefficient of variation; ESRD, end-stage renal disease; GMR, geometric mean ratio; HER, hemodialysis extraction ratio; NA, not applicable; and PK, pharmacokinetic.

^*c*^ Data are presented as geometric mean (geometric CV%) unless otherwise specified. Statistical analyses were performed using an ANOVA model with log-transformed PK parameters as the response variable and group (groups 1–4, group 5 period 1, and group 5 period 2) as a fixed effect. Estimated mean differences and corresponding 90% CIs were back-transformed to obtain GMRs and their associated 90% CIs.

^*d*^ For the pre-dialysis period, geometric mean AUC_0–*t*_ was AUC_0–∞_, as hemodialysis-mediated drug removal truncated the terminal elimination phase and precluded reliable estimation of AUC_0–∞_.

^*e*^ Dosing was after hemodialysis.

Dialysate PK parameters, including hemodialysis extraction ratios (HERs), were evaluated for ceftibuten and ledaborbactam in group 5. For ceftibuten, HER1 (expressed as dialysis clearance method) was 0.23, and HER2 (estimated with AUC ratio method) was 0.43. For ledaborbactam, HER1 and HER2 were 0.38 and 0.33, respectively (Table 4).

### Safety and tolerability

The frequencies of treatment-emergent adverse events (TEAEs) by system organ class and preferred term are summarized in Table 5. Overall, six TEAEs were reported among six participants, including three participants who reported gastrointestinal system organ class TEAEs. Four participants reported mild TEAEs considered by the investigator to be treatment related (diarrhea, soft feces, flatulence, and headache in one participant each), and one participant in group 3 reported a moderate unrelated TEAE (hypertension). No TEAEs led to discontinuation of the study drug; the one study discontinuation (group 5) was due to an unstable hemodialysis access port that was not considered an adverse event.

**TABLE 5:** Summary of treatment-emergent adverse events^*a*^^,*b*,*c*^

System organ class preferred term^*d*^^,*e*^	Renal function group					
	Group 1: normal function (*n* = 9)	Group 2: mild impairment (*n* = 5)	Group 3: moderate impairment (*n* = 6)	Group 4: severe impairment (*n* = 5)	Group 5: ESRD (*n* = 7)	Overall (*N* = 32)
Gastrointestinal disorders	1 (11.1)	1 (20.0)	1 (16.7)	0	0	3 (9.4)
Diarrhea	0	1 (20.0)	0	0	0	1 (3.1)
Feces soft	1 (11.1)	0	0	0	0	1 (3.1)
Flatulence	0	0	1 (16.7)	0	0	1 (3.1)
Nervous system disorders	1 (11.1)	0	0	0	0	1 (3.1)
Headache	1 (11.1)	0	0	0	0	1 (3.1)
Skin and subcutaneous tissue disorders	0	1 (20.0)	0	0	0	1 (3.1)
Dermatitis contact	0	1 (20.0)	0	0	0	1 (3.1)
Vascular disorders	0	0	1 (16.7)	0	0	1 (3.1)
Hypertension	0	0	1 (16.7)	0	0	1 (3.1)

^*a*^ Data include all participants who received at least one dose of ceftibuten-ledaborbactam etzadroxil.

^*b*^ ESRD, end-stage renal disease.

^*c*^ All data are presented as *n* (%). Normal renal function (eGFR ≥90 mL/min), mild impairment (eGFR 60 to <90 mL/min), moderate impairment (eGFR 30 to <60 mL/min), severe impairment (eGFR <30 mL/min), and ESRD (undergoing standard intermittent dialysis).

^*d*^ Adverse events are coded using the Medical Dictionary for Regulatory Activities version 25.1.

^*e*^ Participants were counted only once within each system organ class and preferred term.

No deaths or serious adverse events were reported during the study. No clinically meaningful changes were observed in clinical laboratory values, electrocardiogram (ECG) parameters, or vital signs.

## DISCUSSION

In this phase 1 study, systemic exposures to ceftibuten and ledaborbactam increased progressively, and their CLr decreased, with declining renal function following a single dose of ceftibuten-ledaborbactam etzadroxil. These effects, including up to 3.7- and 4.4-fold increases in the AUC_0–∞_ for ceftibuten and ledaborbactam, respectively, in participants with severe renal impairment, support the need for dosage adjustment in patients with renal impairment.

Urinary PK analyses demonstrated that CLr and Fe decreased with declining renal function for both ceftibuten and ledaborbactam. The geometric mean Fe decreased from 36.5% to 19.4% for ceftibuten and from 54.6% to 36.7% for ledaborbactam across groups 1–4. These findings are consistent with the reduced capacity for renal excretion in participants with impaired renal function. The observed decrease in Fe should therefore be interpreted as a consequence of diminished urinary excretion rather than evidence of altered nonrenal clearance. Although the relative contribution of nonrenal elimination to overall drug disposition may increase as renal excretion declines, this study was not designed to assess or quantify absolute changes in nonrenal clearance.

Exposure to the prodrug ledaborbactam etzadroxil was negligible in plasma and urine across all renal function groups, consistent with rapid and extensive conversion to ledaborbactam. This is in agreement with prior phase 1 data showing that ledaborbactam etzadroxil exposure is <2% of that of ledaborbactam (9, 12, 14). Exposure to ceftibuten and ledaborbactam in participants with normal renal function (group 1) was consistent with that observed in previous phase 1 studies (9, 12, 14). In addition, the unbound fraction of ledaborbactam increased with decreasing renal function. Although the mean ledaborbactam *C*_max_ was slightly lower in participants with moderate renal impairment than in those with mild renal impairment, this isolated observation is likely attributable to interparticipant variability and the small sample size typical of dedicated renal impairment studies. Consistent with this interpretation, regression analysis showed no statistically significant association between ledaborbactam *C*_max_ and eGFR, whereas exposure increased significantly with declining renal function, reflecting reduced drug clearance rather than changes in peak absorption.

In participants with ESRD undergoing intermittent hemodialysis, pre- and post-dialysis PK analyses and HERs demonstrated that both ceftibuten and ledaborbactam were moderately removed to similar extents by intermittent hemodialysis (15). The approximately 50% reduction in AUC_₀–*t*_, observed with pre-dialysis versus post-dialysis, confirms that hemodialysis substantially reduces systemic exposure to both agents. These findings indicate that administration of ceftibuten-ledaborbactam etzadroxil after completion of hemodialysis is preferable to pre-dialysis dosing to maximize systemic drug exposure, with formal dosing recommendations to be established through PK/pharmacodynamic (PD) modeling. The difference between HER1 (0.2) and HER2 (0.4) estimates for ceftibuten reflects differences in methodological approach, with HER1 representing total drug recovery in dialysate relative to dose over the collection period, and HER2 reflecting arteriovenous extraction across the dialyzer during the intradialytic period; both measures are consistent with moderate dialyzability.

A single dose of ceftibuten-ledaborbactam etzadroxil was generally safe and well tolerated in study participants with varying degrees of renal function. The TEAEs observed were predominantly mild gastrointestinal events, consistent with the known tolerability profile of ceftibuten and the combination of ceftibuten-ledaborbactam etzadroxil (9, 12, 14). Despite substantially increased systemic exposure in participants with severe renal impairment and ESRD compared with those with normal renal function, no changes in the safety profile were observed to suggest an exposure-related safety signal following a single dose of ceftibuten-ledaborbactam etzadroxil.

A limitation of this study is the single-dose treatment design, which precluded an assessment of steady-state PK in participants with renal impairment. In addition, no dose adjustment was implemented in this study to normalize exposure between healthy and renally impaired participants. However, these single-dose PK data are suitable for population PK modeling and PK/PD simulations to predict effective renal impairment doses for late-stage efficacy evaluation. Although healthy controls were matched to participants with renal impairment by age, sex, and BMI, demographic differences remained across renal impairment groups, particularly among participants with ESRD, who were younger and predominantly Black or African American. Given the small sample size typical of dedicated renal impairment studies, residual demographic differences could potentially confound between-group comparisons. Nevertheless, renal function is expected to be the primary determinant of the observed PK differences. Finally, although multidose safety was not evaluated in the renally impaired population, multiple-dose studies in healthy participants have demonstrated a favorable safety profile, primarily characterized by mild gastrointestinal adverse events, consistent with the drug class (12). Based on exposure-matching considerations, no clinically meaningful difference in safety is anticipated in renally impaired patients.

In conclusion, this study demonstrated that plasma exposure to both ceftibuten and ledaborbactam increased and CLr decreased with declining renal function following administration of a single dose of ceftibuten-ledaborbactam etzadroxil. Both ceftibuten and ledaborbactam were moderately removed by intermittent hemodialysis. The safety and tolerability profile of ceftibuten-ledaborbactam etzadroxil appeared similar between participants with renal impairment and matched participants with normal renal function, and no safety findings attributable to increased drug exposure were observed in participants with renal impairment. These results support the need for dosage adjustment across the spectrum of renal impairment, including ESRD. The exposure data generated in this study will be incorporated into an updated population PK model to support PK/PD target attainment analyses and inform final dosing recommendations.

## MATERIALS AND METHODS

### Study design

This phase 1, open-label, multicenter, parallel-group study (NCT05488678) was conducted between October 2022 and November 2023 in participants with varying degrees of renal function. The PK, safety, and tolerability of the inactive prodrug ledaborbactam etzadroxil, its active moiety ledaborbactam, and ceftibuten were evaluated following oral dosing of ceftibuten-ledaborbactam etzadroxil.

Sample size was not based on formal hypothesis testing or statistical power calculations. Instead, approximately five to eight participants per renal function group were considered sufficient to characterize the PK of ceftibuten and ledaborbactam across the spectrum of renal function. This sample size is consistent with established practice for dedicated renal impairment studies (16, 17). Participants were enrolled into groups 1–4 based on eGFR determined using the Chronic Kidney Disease Epidemiology Collaboration equation (18). Absolute eGFR (mL/min) was calculated from the indexed value (mL/min/1.73 m^2^) by multiplying by the individual’s body surface area and dividing by 1.73 m^2^ to reflect individual renal function for purposes of group assignment (18). Group 1 consisted of age-, sex-, and BMI-matched control participants with normal renal function (eGFR ≥90 mL/min). Group 2 consisted of participants with mild renal impairment (eGFR 60 to <90 mL/min), group 3 consisted of participants with moderate renal impairment (eGFR 30 to <60 mL/min), and group 4 consisted of participants with severe renal impairment (eGFR <30 mL/min) who were not undergoing hemodialysis. Group 5 consisted of participants with ESRD undergoing intermittent hemodialysis.

Participants in groups 1–4 received a single oral dose of ceftibuten-ledaborbactam etzadroxil (600 mg/600 mg) on day 1. Participants in group 5 were evaluated to assess the effect of intermittent hemodialysis on PK during hemodialysis and off hemodialysis. In group 5, participants received an oral dose of ceftibuten-ledaborbactam etzadroxil (600 mg/600 mg) on day 1 of period 1, administered prior to a hemodialysis session, with hemodialysis initiated 2 h post-dose. Following a 1- to 2-week washout period, participants received a second oral dose of ceftibuten-ledaborbactam etzadroxil (600 mg/600 mg) on day 1 of period 2, administered 15–30 min after completion of a hemodialysis session.

### Participants

Adult men and women were eligible if 18–82 years of age with a BMI of 18.5–40.0 kg/m^2^ and a minimum weight of 45 kg. Women were required to be nonpregnant and nonlactating and, if not postmenopausal, to use an accepted form of contraception from the time of informed consent through 90 days after the last dose. All participants were required to have blood pressure and clinical laboratory values within protocol-defined ranges at screening and on day −2. Key exclusion criteria for all participants included a history of hypersensitivity to cephalosporins, penicillins, or other β-lactam antibiotics; congenital or acquired immunodeficiency; a major adverse cardiovascular event within 1 year prior to day 1; positive drug or alcohol screening; a hepatitis B or C infection or human immunodeficiency virus positivity; using acid-reducing agents such as proton pump inhibitors (within 14 days prior to admission), H2-antagonists (within 5 days prior to admission), or over-the-counter antacids (within 24 h prior to admission); or receipt of coronavirus disease 2019 vaccine within 14 days prior to dosing, or known exposure to or infection with coronavirus disease 2019 within 14 days prior to the check-in day.

For inclusion in group 1 (matched-control healthy participants with normal renal function [≥90 mL/min]), participants were required to be in good health, with no clinically significant findings on medical history, physical examination, ECG, vital signs, or clinical laboratory evaluations. Each matched-control healthy participant was demographically matched to at least one enrolled renally impaired participant by age (±10 years), BMI (±20%; based on screening data), and sex at birth. Key exclusion criteria included use of any prescription or nonprescription medications or products within 14 days prior to dosing, with the exception of contraceptive agents and acetaminophen at doses ≤3 g/day; QT interval corrected for heart rate using Fridericia’s method >450 ms in males or >470 ms in females; or history or evidence of long QT syndrome.

For participants with renal impairment (groups 2–5), key eligibility criteria included stable renal function (groups 2–4 only) for at least 4 weeks prior to screening and a stable concomitant medication and/or treatment regimen within 14 days prior to administration of study drug on day 1. An exception was made for acetaminophen, which was permitted at doses ≤3 g/day. Exclusion criteria for groups 2–5 included a currently functioning or scheduled kidney transplant, current or prior hemodialysis (groups 2–4 only), or a QT interval corrected for heart rate using Fridericia’s method >480 ms at screening or check-in.

### Assessments

Plasma PK parameters included *C*_max_, AUC_0–*t*_, AUC_0–∞_, *t*_1/2_, and CL/F (ledaborbactam etzadroxil and ceftibuten only), as well as AUC during dialysis (ESRD participants on hemodialysis). Two blood samples were collected at each nominal time point for ceftibuten and ledaborbactam/ledaborbactam etzadroxil measurement. For groups 1–4, PK samples were collected pre-dose (within 30 min prior to dosing) and at 0.5, 1, 1.5, 2, 2.5, 3, 4, 6, 8, 12, 16, 24, 48, 72, 96, and 120 h post-dose. For group 5, blood samples were collected in both period 1 and period 2. Sampling was performed pre-dose (within 30 min prior to dosing) and at 0.5, 1, 1.5, 2, 2.5, 3, 4, 5, 6, 7, 8, 12, 16, 24, 48, and 72 h post-dose; samples beyond 48 h were not collected if a scheduled dialysis session occurred prior to the planned sampling time. In period 1 only, arterial and venous blood samples were collected immediately prior to the initiation of dialysis and hourly during dialysis. Arterial-line samples were obtained pre-hemodialysis, and venous-line samples were taken post-hemodialysis. Blood samples collected for groups 1–5 at 2 and 12 h post-dose (pre- and post-dialysis in group 5) were also analyzed for unbound ledaborbactam. For the pre-dialysis period (group 5), geometric mean AUC_0–*t*_ was reported instead of AUC_0–∞_, as hemodialysis-mediated drug removal truncated the terminal elimination phase and precluded reliable estimation of AUC_0–∞_.

Urinary PK parameters included CLr and the Fe. Urine was collected for PK assessment from groups 1–4 on days 1, 2, 3, 4, 5, and 6 over the intervals 0–4, 4–8, 8–12, 12–24, 24–48, 48–72, 72–96, and 96–120 h post-dose.

Dialysate parameters included the amount of drug recovered in dialysate (*A*_*d*_), HER for the fraction of drug removed during a dialysis session expressed as a percentage according to *A*_*d*_/dose (HER1), and HER2, calculated as (AUC of arterial concentrations during dialysis [AUC_in_] − AUC of venous concentrations during dialysis [AUC_out_])/AUC_in_. Dialysate was collected in group 5 during period 1. Samples were collected at dialysis initiation and every 30 min thereafter throughout the approximately 4-h dialysis session for a maximum of nine samples per participant.

Plasma, urine, and dialysate PK samples were analyzed for ceftibuten, ledaborbactam, and ledaborbactam etzadroxil using validated bioanalytical methods (9, 12, 14). Analytes were extracted from plasma and urine using protein precipitation, whereas ceftibuten in dialysate was quantified following dilution. All analytes were quantified by tandem mass spectrometry with ceftibuten (as *cis*-ceftibuten) in positive ion mode across all matrices and with ledaborbactam and ledaborbactam etzadroxil in negative ion mode across all matrices. Isotopically labeled internal standards were used for all analytes.

Plasma protein binding of ledaborbactam was assessed using a rapid equilibrium dialysis method with respective measures of total drug to determine protein binding.

Safety was assessed by monitoring TEAEs, coded using the Medical Dictionary for Regulatory Activities version 25.1, and by review of clinical laboratory results, ECG parameters, vital signs, and physical examinations.

### PK and statistical analyses

The safety set, which consisted of all participants who received at least one dose of ceftibuten-ledaborbactam etzadroxil, was used to summarize safety data and baseline characteristics. The PK set, which consisted of all participants who received at least one dose of ceftibuten-ledaborbactam etzadroxil and had at least one evaluable PK sample for any analyte, was used for concentration summaries, PK parameter analyses, and statistical evaluations.

Plasma concentrations of ceftibuten, ledaborbactam, and ledaborbactam etzadroxil below the quantifiable limit (BQL) were imputed as zero to calculate arithmetic mean concentrations and as the lower limit of quantification/2 to calculate geometric means and percent coefficient of variation of the geometric mean. Descriptive statistics were used to summarize plasma concentrations by renal function group (groups 1–5) and study period (group 5, periods 1 and 2) at each scheduled time point. Mean concentrations were reported as zero when all values were BQL; no additional descriptive statistics were provided in such cases. When the calculated mean was less than the lower limit of quantification, values were reported as noncalculable.

Plasma PK parameters for ceftibuten, ledaborbactam, and ledaborbactam etzadroxil were estimated from individual concentration-time profiles using noncompartmental methods and actual sampling times. AUCs were calculated using the linear-up/log-down trapezoidal method with uniform weighting. In PK analyses, BQL values occurring before the first quantifiable concentration were set to zero, whereas BQL values occurring after the first quantifiable concentration were treated as missing. Isolated quantifiable concentrations between BQL values or concentrations occurring after two or more consecutive BQL values were treated as missing at the discretion of the investigator.

Urine PK parameters were derived from measured urine concentrations and recorded weights. Urine volumes were estimated assuming a mean density of 1.01 g/mL (volume [mL] = weight [g]/1.01 g/mL). Missing values were treated as missing, and BQL values were set to zero. If any missing value was present for a participant, cumulative PK parameters were not calculated for that participant. Descriptive statistics were used to summarize urinary PK parameters for ledaborbactam etzadroxil, ledaborbactam, and ledaborbactam etzadroxil-derived material (defined as the sum of molar-equivalent concentrations of ledaborbactam etzadroxil and ledaborbactam), as well as ceftibuten, by renal function group (groups 1–4) at each scheduled interval. Total ledaborbactam etzadroxil-derived material was calculated using molecular weights of 391.23 g/mol (ledaborbactam etzadroxil) and 263.10 g/mol (ledaborbactam).

Dialysate PK parameters were derived from measured dialysate concentrations and volumes. Missing values were treated as missing, and BQL values were set to zero. If any missing value occurred for a participant, cumulative dialysate PK parameters were not calculated. Dialysate AUCs were calculated using the linear-up/log-down trapezoidal method.

Statistical analyses and data reporting were performed using SAS for Windows version 9.4 or higher (SAS Institute, Inc., Cary, NC, USA). PK parameters were calculated primarily using Phoenix WinNonlin version 8.1 or higher (Certara, L.P., Radnor, PA, USA). Additional PK computations were performed in SAS, as appropriate.

## AFTER EPUB

*[This article was published on 28 September 2026 with footnote errors in Tables 1 and 4. The tables were corrected in the current version, posted on 30 September 2026.]*

## SUPPLEMENTAL MATERIAL

Supplemental material aac.00860-26-s0001.pdf Tables S1 and S2.

ASM does not own the copyrights to Supplemental Material that may be linked to, or accessed through, an article. The authors have granted ASM a non-exclusive, world-wide license to publish the Supplemental Material files. Please contact the corresponding author directly for reuse.
