# Supplementary material for: Pharmacokinetics, safety, and tolerability of ceftibuten-ledaborbactam etzadroxil in participants with renal impairment
Source: Antimicrob Agents Chemother. 2026 Sep 28;70(11):e00860-26. doi: 10.1128/aac.00860-26 (PMC13731173; doi:10.1128/aac.00860-26)
Supplement: Supplementary file 1 [file aac.00860-26-s0001.pdf]

Table S1 Geometric mean (geometric CV%) plasma PK parameters for ledaborbactam etzadroxil across renal function groups 1–4[a,b,c]

Renal function group (eGFR range, [mL/min])	C_{max} (ng/mL)	$t_{1/2}$ (h)	$AUC_{(0-\infty)}$ (h·ng/mL)	CL/F (L/h)	Fe (%)	CLr (L/h)
Group 1<br>Normal function (≥90)<br>*n* = 9	746<br>(101)	4.2<br>(46.3)	931<br>(76.5)	644<br>(76.5)	0.004<br>(48.6)	0.02<br>(101)
Group 2<br>Mild impairment<br>(60–<90)<br>*n* = 5	1100<br>(81.6)	4.6<br>(5.2)	1460<br>(32.7)	411<br>(32.7)	0.003<br>(62.5)	0.01<br>(55.2)
Group 3<br>Moderate impairment<br>(30–<60)<br>*n* = 6	790<br>(119)	5.1<br>(38.3)	1370<br>(59.6)	439<br>(59.6)	0.002<br>(55.9)	0.008<br>(120)
Group 4<br>Severe impairment (<30)<br>*n* = 5	1340<br>(38.2)	4.5<br>(41.4)	1230<br>(28.0)	487<br>(28.0)	0.001<br>(31.8)	0.006<br>(43.5)

[a]Data include all participants who received at least one dose of ceftibuten/ledaborbactam etzadroxil and had at least one evaluable PK sample of any of the assessed analytes.
[b]All data are presented as geometric mean (geometric CV%).
[c]$AUC_{0-\infty}$, area under the concentration-time curve from time zero to infinity; CL/F, apparent clearance; CLr, renal clearance; C_{max}, maximum observed plasma concentration; CV, coefficient of variation; eGFR, estimated glomerular filtration rate; Fe, fraction of dose excreted unchanged into urine; PK, pharmacokinetic; $t_{1/2}$, terminal elimination half-life.

Table S2 Geometric mean (geometric CV%) urine PK parameters for ceftibuten and ledaborbactam across renal function groups 1–4[a,b,c]

Renal function group (eGFR range, [mL/min])	Ceftibuten Fe (%)	Ceftibuten CLr (L/h)	Ledaborbactam Fe (%)	Ledaborbactam CLr (L/h)
Group 1<br>Normal function (≥90)<br>Ceftibuten/ledaborbactam *n* = 9/7	36.5 (30.1)	2.16 (28.3)	54.6 (28.8)	3.04 (28.9)
Group 2<br>Mild impairment (60–<90)<br>Ceftibuten/ledaborbactam *n* = 5/5	25.0 (41.3)	1.09 (31.6)	44.1 (40.2)	1.70 (44.8)
Group 3<br>Moderate impairment (30–<60)<br>Ceftibuten/ledaborbactam *n* = 5/6	33.7 (48.5)	0.893 (76.4)	52.4 (39.0)	1.56 (105)
Group 4<br>Severe impairment (<30)<br>Ceftibuten/ledaborbactam *n* = 5/5	19.4 (57.2)	0.310 (61.4)	36.7 (50.8)	0.471 (56.6)

[a]Data include all participants who received at least one dose of ceftibuten/ledaborbactam etzadroxil and had at least one evaluable PK sample of any of the assessed analytes.
[b]All data are presented as geometric mean (geometric CV%).
[c]CLr, renal clearance; CV, coefficient of variation; eGFR, estimated glomerular filtration rate; Fe, fraction of dose excreted unchanged into urine; PK, pharmacokinetic.
